# The role of ^18^F-fluorodeoxyglucose-positron emission tomography/computed tomography in the differential diagnosis of pericardial disease

**DOI:** 10.1038/s41598-020-78581-y

**Published:** 2020-12-09

**Authors:** Cheol Won Hyeon, Hyun Kyung Yi, Eun Kyoung Kim, Sung-Ji Park, Sang-Chol Lee, Seung Woo Park, Jae K. Oh, Joon Young Choi, Sung-A Chang

**Affiliations:** 1 Division of Cardiology, Department of Medicine, Samsung Medical Center, Sungkyunkwan University School of Medicine, 81 Irwon-ro, Gangnam-gu, Seoul, 06351 Republic of Korea; 2 Department of Nuclear Medicine, Samsung Medical Center, Sungkyunkwan University School of Medicine, 81 Irwon-ro, Gangnam-gu, Seoul, 06351 Republic of Korea; 3 Heart Vascular Stroke Imaging Center, Heart Vascular Stroke Institute, Samsung Medical Center, Sungkyunkwan University School of Medicine, Seoul, Republic of Korea; 4 Department of Cardiovascular Medicine, Mayo Clinic College of Medicine, Rochester, USA

**Keywords:** Cardiology, Medical research

## Abstract

This study aimed to assess the role of ^18^F-fluorodeoxyglucose-positron emission tomography/computed tomography (^18^FDG-PET/CT) in the differential diagnosis of pericardial disease. The diagnosis is often troublesome because pericardial fluid analysis or biopsy does not always provide answers. ^18^FDG-PET/CT can visualize both inflammation and malignancy and offers a whole-body assessment. Patients who visited the Pericardial Disease Clinic of Samsung Medical Center with an ^18^FDG-PET/CT order code were extracted. Exclusion criteria were as follows: (1) the purpose of the differential diagnosis was not pericardial disease; (2) the patient had a known advanced-stage malignancy; (3) the patient already have confirmative diagnosis using a serology, pericardial effusion analysis or biopsy. The analysis included 107 patients. The most common final diagnosis was idiopathic (n = 46, 43.0%), followed by tuberculosis (n = 30, 28.0%) and neoplastic (n = 11, 10.3%). A maximum standardized uptake value (SUVmax) ≥ 5 typically indicates tuberculosis or neoplastic pericarditis except in just one case of autoimmune pericarditis); especially all of the SUVmax scores ≥ 10 had tuberculosis. The diagnostic yield of pericardial biopsy was very low (10.2%). Interestingly, all of the pericardium with an SUVmax < 4.4 had nondiagnostic results. In contrast, targeted biopsies based on ^18^FDG uptake demonstrated a higher diagnostic yield (38.7%) than pericardium. The sensitivity of ^18^FDG-PET/CT was 63.6%. The specificity was 71.9%. The positive predictive value was 20.6%. The negative predictive value 94.5%, and the accuracy was 71.0% for excluding malignancy based upon the FDG uptake patterns. It is possible to explore the differential diagnosis in some patients with difficult pericardiocentesis or pericardial biopsy in a noninvasive manner using on the SUVmax or uptake patterns. In addition, the biopsy strategy depending on ^18^FDG uptake is helpful to achieve biopsy more safely and with a higher yield. ^18^FDG-PET may enhance the diagnostic efficacy in patients with pericardial disease.

## Introduction

Pericardial diseases are relatively common in clinical practice and may have varying presentations either as isolated disease or as a manifestation of a systemic disorder^1–3^. The etiology of pericardial disease of various causes is not only limited to disease in the pericardium itself; a systemic approach during the differential diagnosis is therefore frequently required. The amount of pericardial effusion and hemodynamic impact can be easily assessed using echocardiography^4^; however, limited information exists regarding adjacent organs or the full range of the pericardium itself due to the limited echocardiographic window. It is also difficult to differentiate the properties of effusion by echocardiography alone. Therefore, a multimodal imaging approach has been suggested using computed tomography/magnetic resonance imaging (CT/MRI).

A pericardial fluid analysis is essential to characterize pericardial effusion; however, pericardiocentesis is limited based on its location and the amount of pericardial effusion. Pericardial tissue can be obtained through a surgical approach, but the information is frequently nondiagnostic, and the process itself can be technically challenging due to constriction or adhesions. The presence of an underlying disease that causes pericardial effusion determines the diagnosis and treatment of pericardial disease^5^. Therefore, noninvasive functional imaging in pericardial disease is helpful to discriminate the presence of inflammation or malignancy not only in the pericardium but also in other organs. As a result, ^18^F-fluorodeoxyglucose-positron emission tomography/computed tomography (^18^FDG-PET/CT) is a good candidate to assess pericardial disease, which includes inflammatory or malignant disease, in a single examination^6^. It has been reported that pericardial SUV_max_ predict the reversibility of transient constrictive pericarditis^7^. However, research into the clinical implications of ^18^FDG-PET/CT in pericardial disease is very limited.

In this study, we investigated the clinical implications of ^18^FDG-PET/CT in real-world clinics in patients with pericardial disease.

## Results

**Figure 1:**
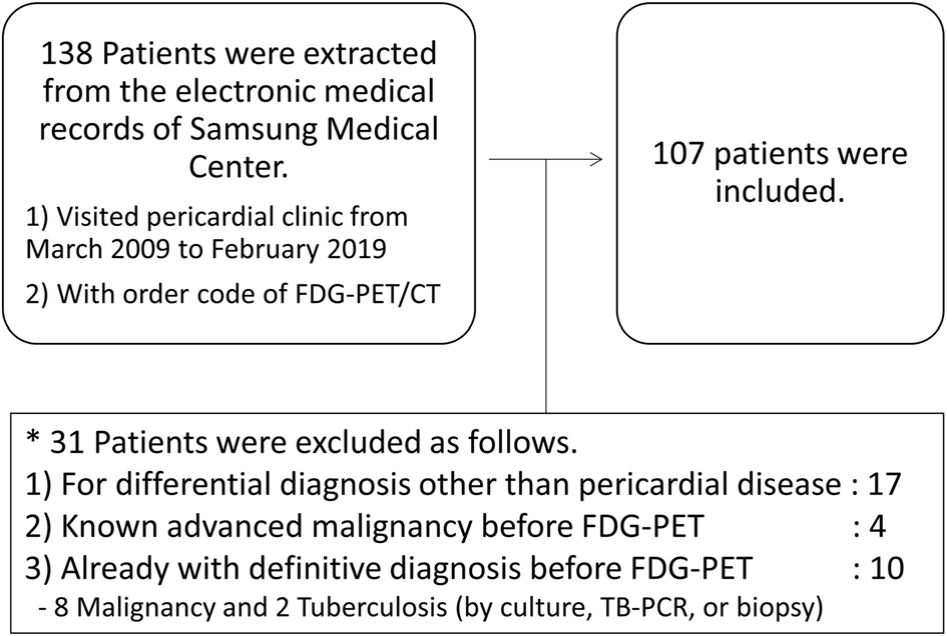
The study population.

One hundred and nine patients were finally analyzed. The patient selection process is depicted in Fig. 1, including the process for excluding patients. Out of the total 109 patients, 42 (39.2%) had pericardial effusion at their initial presentation and underwent pericardiocentesis or pericardial biopsy before the ^18^FDG-PET/CT was performed, and the pericardial fluid or tissue analysis was nondiagnostic. Thirty-one patients (29.0%) had no or only minimal pericardial effusion with constrictive physiology or a thickened pericardium. The rest of the patients (n = 34, 31.8%) presented with a small amount of pericardial effusion or inadequate localization for pericardiocentesis.

The patients’ general characteristics and past medical history are summarized in Table 1. The median age of the study population was 60.1 ± 15.4 years, and the male sex was dominant. Any underlying disease that could affect the pericardial disease was reviewed. The percentages of patients with a malignancy or tuberculosis were 23.4% and 15.0%, respectively; however, all of them had already been treated, and there was no evidence of a recent recurrence.

**Table 1:** General characteristic and past medical history.

Characteristics	
Age (year)	60.1 (± 15.4)
Male gender	66 (61.7%)
Hypertension	33 (30.8%)
Diabetes mellitus	16 (15.0%)
Dyslipidemia	17 (15.9%)
**Previous medical history of**	
Chronic kidney disease	5 (4.7%)
Open heart surgery	16 (15.0%)
Malignancy (cure or complete remission)	25 (23.4%)
Radiation therapy	13 (12.1%)
Tuberculosis	16 (15.0%)
Autoimmune disease	0 (0.0%)

Data are presented as number (%) or mean (± standard deviation).

After an integrated diagnostic work-up and a follow-up period lasting more than 6 months, the final diagnosis was made by an attending physician (Table 2). Idiopathic pericarditis was the most common finding (43.0%), while tuberculosis accounted for 28.0% of cases, and 10.3% were neoplastic. Details of neoplastic pericarditis is as follows; mesothelioma (n = 3), angiosarcoma (n = 1), hemangioendothelioma (n = 1) and metastatic carcinoma (n = 6, from lung (n = 2), thymus, thyroid, stomach, breast). Less common causes included radiation, post-cardiac injury syndrome, parasitic infection, autoimmune pericarditis, and traumatic hemopericardium.

**Table 2:** Final diagnosis.

Diagnosis	Number (%)
Idiopathic pericarditis	46 (43.0)
Tuberculous pericarditis	30 (28.0)
Neoplastic pericarditis	11 (10.3)
Post-cardiac injury syndrome	9 (8.4)
Radiation-induced pericarditis	7 (6.5)
Parasite infection	2 (1.9)
Autoimmune disease	1 (0.9)
Traumatic hemopericardium	1 (0.9)

**Figure 2:**
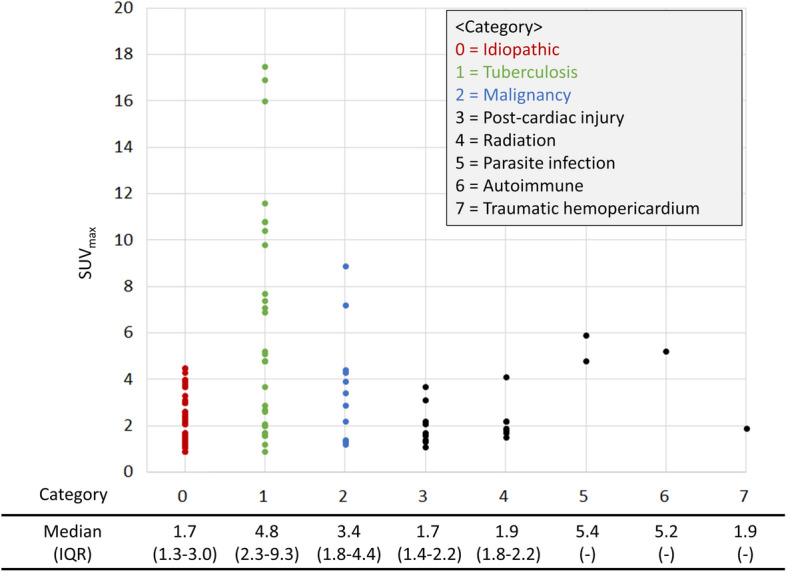
A scatter plot of the pericardial SUV_max_ scores according to the final diagnosis. [Central illustration].

The pericardial SUV_max_ was analyzed according to the final diagnosis (Fig. 2). None of the idiopathic pericarditis cases showed a pericardial SUV_max_ value > 5. Tuberculosis had the highest variation in SUV_max_ scores. Malignancy had a higher median pericardial SUV_max_ value than idiopathic pericarditis, but none of the scores were > 10.

**Figure 3:**
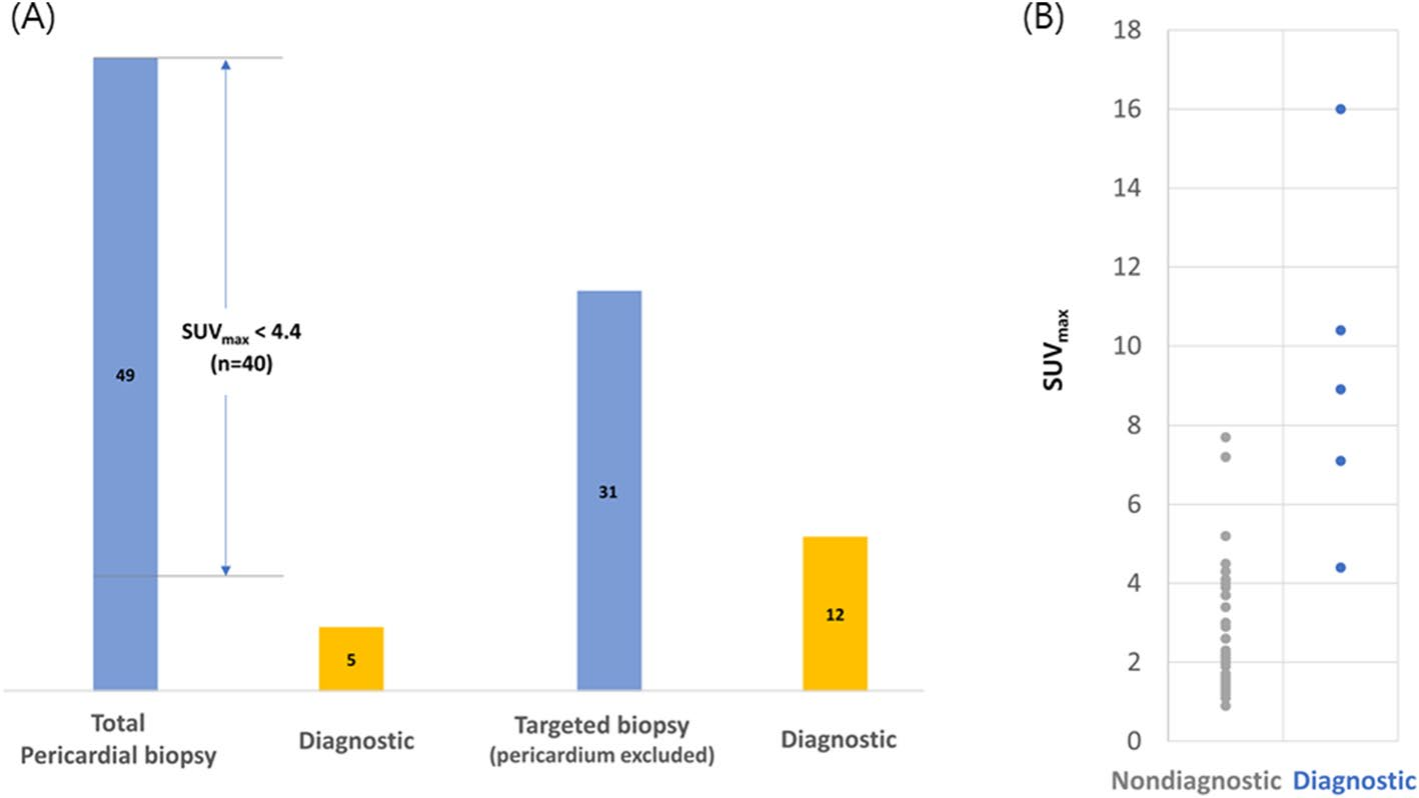
(**A**) A scatter plot of the pericardial SUV_max_ scores with the diagnostic and nondiagnostic results of the pericardial biopsy. (**B**) The number of patients who underwent total pericardial biopsy and diagnostic pericardial biopsy/total targeted biopsy (pericardium excluded) and diagnostic targeted biopsy.

Pericardial biopsy was finally performed in 49 patients, but the diagnostic yield was very low (10.2%) (Fig. 3A left). Pericardial SUV_max_ in nondiagnostic vs. diagnostic pericardial biopsy is demonstrated in in Fig. 3B. In 31 patients, an additional targeted biopsy based upon the ^18^FDG-PET/CT findings was performed at 46 biopsy sites other than the pericardium. These sites demonstrated a higher diagnostic yield (38.7%) than the pericardium (Fig. 3A right). The most common targeted biopsy site was a mediastinal mass or a lymph node (39.1%), followed by the supraclavicular lymph nodes and the cervical lymph nodes (Table 3).

**Table 3:** Targeted biopsy site by FDG-PET.

Targeted biopsy site	Number (%)
Mediastinal mass or lymph nodes	18 (39.1)
Supraclavicular lymph nodes	5 (10.9)
Cervical lymph nodes	5 (10.9)
Thyroid	4 (8.7)
Pleura	4 (8.7)
Axillary lymph nodes	2 (4.3)
Lung	2 (4.3)
Breast	2 (4.3)
Colon and rectum	2 (4.3)
Liver	2 (4.3)

Table 4 shows the presumptive diagnosis based upon the ^18^FDG-PET/CT and the distribution of final diagnoses. Among the cases that suggested malignancy, only 3 of 7 had malignant disease. Four of 73 cases reported as benign were finally diagnosed as malignancies. On the basis of uptake pattern, the negative predictive value of ^18^FDG-PET/CT to exclude malignancy in pericardial disease was more than 94.5% (69/73).

**Table 4:** PET/CT interpretation for the pericardial lesions and final diagnosis.

	Probable benign (n = 73)	Probable malignancy (n = 7)	Equivocal (n = 27)
Idiopathic pericarditis	35	0	11
Tuberculous pericarditis	16	3	11
Neoplastic pericarditis	4	3	4
Post-cardiac injury syndrome	9	0	0
Radiation-induced pericarditis	7	0	0
Parasite infection	0	1	1
Autoimmune disease	1	0	0
Traumatic hemopericardium	1	0	0

Difference in the shape and properties of ^18^FDG accumulation between tuberculous pericarditis and neoplastic disease was also analyzed. Images were categorized as no uptake, focal uptake, diffuse homogeneous uptake, diffuse heterogenous uptake and there was no significant difference between groups (Table 5).

**Table 5:** Difference in the shape and properties of ^18^FDG accumulation between tuberculous pericarditis and neoplastic disease.

	Tuberculous pericarditis (n = 30)	Neoplastic pericarditis (n = 11)
Normal uptake	5 (17%)	1 (9%)
Focal uptake	2 (7%)	1 (9%)
Diffuse homogeneous uptake	4 (13%)	2 (19%)
Diffuse heterogenous uptake	19 (63%)	7 (63%)

## Discussion

The role of ^18^FDG-PET/CT in pericardial disease is not clearly established, although the 2015 European Society of Cardiology guidelines^8^ recommend ^18^FDG-PET/CT in special clinical situations with probable autoimmune disease (large vessel arteritis and sarcoidosis) or neoplasms.

^18^FDG-PET/CT is indicated to depict the metabolic activity of pericardial disease. In cases with a diagnosed solid cancer or lymphoma, the pericardial uptake of the ^18^FDG tracer is indicative of malignant pericardial involvement, thus providing essential information on the staging and assessment of the patient’s therapeutic response^9^. The uptake is usually intense and is often associated with a focal soft tissue mass^10^. In particular, tuberculous pericarditis was reported to yield higher ^18^FDG uptakes than idiopathic forms in a small study with only five cases of tuberculosis^11^. However, the role of ^18^FDG-PET/CT in the differential diagnosis of cases that initially presented as pericardial disease remains challenging^9^.

In this study, ^18^FDG-PET/CT was performed as a second-line diagnostic imaging study for the further evaluation of pericardial disease after pericardial effusion analysis or other imaging study (echocardiography and/or CT). Although the diagnostic yield to differentiate pericardial disease using ^18^FDG-PET alone was not perfect, it was helpful in clinical practice as follows: First, ^18^FDG-PET/CT aided in differentiating pericardial tuberculosis. Annual tuberculosis incidence (per 100,000 population) of East Asian Country including China and South Korea are still 25–99 per 100,000 population^12^, therefore tuberculous pericarditis is still important cause of pericarditis. Furthermore, clinical presentation of tuberculous pericarditis in immunocompetent hosts is various clinical presentation from asymptomatic pericardial effusion to acute pericarditis or constrictive pericarditis, differential diagnosis for tuberculous pericarditis is always critical because of high rate of complication when untreated^13^.

**Figure 4:**
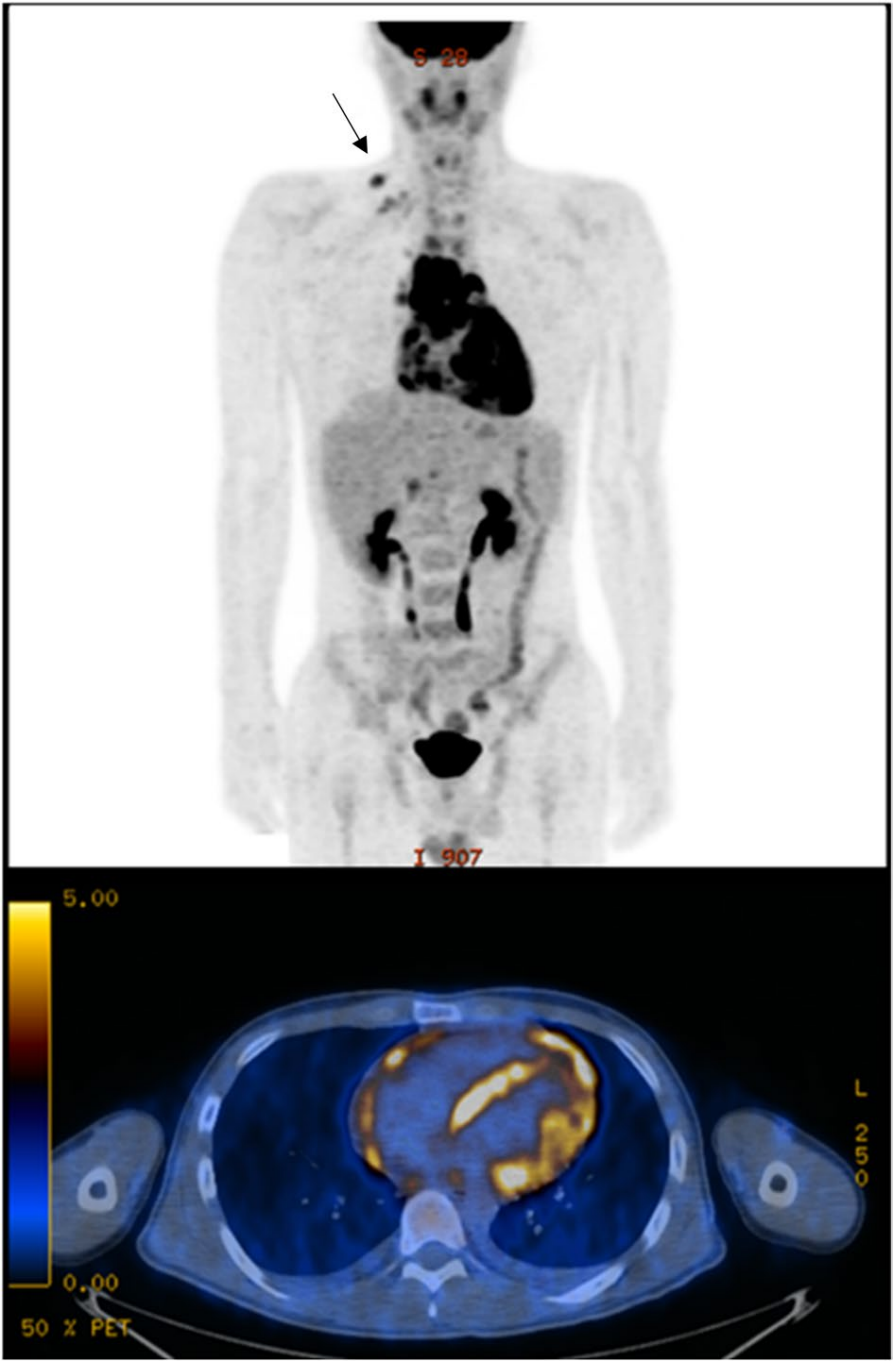
^18^FDG-PET image of a 21-year-old male patient. Tuberculous pericarditis was diagnosed via biopsy right supraclavicular lymph node with increased FDG uptake (black arrow), instead of initially planned pericardial biopsy.

All cases with a pericardial SUV_max_ value that was > 9 had tuberculous pericarditis. Furthermore, none of the tuberculous pericarditis cases were interpreted as suggestive of malignancy. Therefore, if the pericardial SUV_max_ is very high and the ^18^FDG uptake pattern does not suggest a typical malignancy, the possibility of tuberculous pericarditis will be increased. However, tuberculous pericarditis may also present with a low pericardial SUV_max_ value, so a low SUV_max_ does not rule out the diagnosis of tuberculous pericarditis. This trend can be explained by the unique nature of tuberculous pericarditis, which has various clinical presentations in immunocompetent patients^13^ depending on whether they are in the subacute or chronic inflammatory stage. Second, ^18^FDG-PET/CT was helpful to determine the best biopsy site for further evaluations. The diagnostic yield of pericardial biopsy is low and frequently non-diagnostic (the most common finding is chronic inflammation) in clinical practice^14,15^, and our data revealed similar outcomes. Extracardiac tissue biopsy in locations other than the pericardium is often helpful in the diagnosis. However, all surgical procedures carry potential risks, and multiple non-diagnostic findings can be found upon CT imaging without PET images. Extracardiac biopsy targeted by ^18^FDG-PET/CT findings demonstrated a better diagnostic yield than pericardial biopsy and was critical in deciding the best way to manage the patients’ disease. For example, one patient who presented with a fever and pericarditis with a thickened pericardium had multiple lymph node uptake on ^18^FDG-PET/CT. A right supraclavicular lymph node biopsy was easily assessible with local anesthesia. The pathology report of that lymph node indicated caseous necrosis, so the patient was treated for tuberculosis, and the pericardial biopsy was canceled (Fig. 4). In addition, pericardial biopsy results in patients with a low pericardial SUV_max_ < 4 were reported as non-specific inflammatory findings. These results suggest that ^18^FDG-PET can be helpful to avoid unnecessary pericardial biopsies with a high specificity.

The limitations of ^18^FDG-PET/CT have been clearly demonstrated in previous studies^9^ and were found to be similar in our study. Although Shao et al. reported that the SUV_max_ could distinguish malignancies from benign tissue^16^, their study only included patients with tumorous conditions, which makes it a very different study from our data that included patients presenting with pericardial disease of an unknown etiology. In our study, both malignant- and benign-appearing tissues had a large amount of overlap both in the pericardial SUV_max_ score and the tissue uptake patterns. Various pericardial diseases have a similar area of pericardial SUV_max_ scores, especially from 1.0–5.0, which is non-specific (Fig. 2). The tissue uptake pattern was also confusing, especially in malignancies, which have no diagnostic value for differentiation. Moreover, ^18^FDG-PET/CT is expensive and carries a potential amount of radiation exposure when used routinely for pericardial disease. Therefore, our study results should be interpreted with caution and only carefully applied to patients who have trouble with conventional, noninvasive diagnostic methods and who require invasive procedures, such as pericardial or other tissue biopsies.

This study contains the largest dataset of ^18^FDG-PET used in the differential diagnosis of pericardial disease in real clinical practice to date. Although this was a retrospective observational study, it suggests that the pericardial SUV_max_ obtained using ^18^FDG-PET could be helpful to differentiate a high probability of pericardial tuberculosis and also to select a biopsy site that will produce a better yield when compared to conventional approaches, including echocardiography and CT alone. Pericardial disease is commonly comorbid with systemic disease and is not limited to the pericardium. One unique aspect of ^18^FDG-PET as a functional imaging modality is its use in the localization of multi-organ involvement in systemic disease.

## Conclusion

^18^FDG-PET is a noninvasive imaging tool that can visualize the pericardium and any affected organs during a single examination. In patients who require further evaluations, such as a pericardial biopsy, the ^18^FDG-PET can be helpful in selecting the biopsy site with the best diagnostic yield. Very high pericardial SUV_max_ values are helpful when differentiating tuberculous pericarditis from other diseases; however, malignancy cannot be excluded when the pericardial SUV_max_ is < 9. Therefore, ^18^FDG-PET can be helpful to diagnose the cause of pericardial disease in selected patients.

## Methods

### Study population

Patients who visited our pericardial disease clinic from March 2009 to February 2019 were reviewed for inclusion in this study. We retrieved the records of patients who underwent ^18^FDG-PET/CT from our electrical record system database. The following exclusion criteria were applied for the recruited population; (1) the purpose of ^18^FDG-PET/CT was to investigate another disease; (2) a definitive diagnosis was confirmed before ^18^FDG-PET/CT; or (3) the patient already have confirmative diagnosis using a serology, pericardial effusion analysis or biopsy.

All methods were carried out in accordance with relevant guidelines and regulations. The Institutional Review Board of Samsung Medical Center approved the study protocol, and the requirement for written informed consent was waived.

### Data collection

Age, sex, height, body weight, body mass index, and past medical history (including hypertension, diabetes, dyslipidemia, chronic kidney disease, cardiocerebrovascular accident, cardiac procedures, open heart surgery, a previous history of malignancy, previous radiation therapy, and a history of tuberculosis) were reviewed using electronic medical records. Chronic kidney disease was defined when a patient was on dialysis or the serum creatinine level remained ≥ 2.0 mg/dl. Cardiocerebrovascular accident was defined as acute coronary syndrome, myocardial infarction, or stroke. Cardiac procedures included percutaneous coronary interventions, radiofrequency catheter ablation, intracardiac device implantation (such as implantable cardioverter-defibrillator, pacemaker, or cardiac resynchronization therapy), and pericardiocentesis.

### Imaging acquisition of ^18^FDG PET/CT

Image acquisition was performed as previously described^7^. To decrease physiological uptake in the myocardium, preparation of the patients included fasting for at least 12 h before and a low-carbohydrate diet for more than 24 h before the PET/CT examination. Only oral hydration with glucose-free water was allowed during the fasting period. After confirmation of a normal blood glucose level in the peripheral blood (< 150 mg/dl), the patients received an intravenous injection of ^18^FDG (5 MBq/kg) and then rested for approximately 60 min before undergoing scanning by two dedicated PET/CT scanners (Discovery STe or Discovery LS, GE Healthcare, Milwaukee, WI, USA). Immediately after the non-contrast enhanced CT scan (140 keV, 30–170 mAs with AutomA mode, and section width = 3.75 mm for STe; 140 keV, 40–120 mAs adjusted to body weight and section width = 5 mm for LS), an emission PET scan was obtained from the thigh to the head at baseline or for the thorax after steroid therapy with a 2.5-min acquisition time per frame using the three-dimensional mode for the STe and a 4-min acquisition time per frame in two-dimensional mode for the LS. Attenuation-corrected PET images (voxel size = 4.3 × 4.3 × 3.9 mm for the Discovery LS, 3.9 × 3.9 × 3.3 mm for the STe) were reconstructed using CT data and ordered-subset expectation maximization algorithms (20 subsets and 2 iterations for the STe; 28 subsets and 2 iterations for the LS). The standardized uptake values (SUVs) were derived from the injected dose of ^18^FDG and the patient’s body weight. Commercial software (Advantage Workstation version 4.4, GE Healthcare) was used to accurately coregister the separate CT and PET scan data and to measure the maximum SUV (SUV_max_) value of the pericardium.

### Clinical follow-up data analysis

Clinical follow-up data were categorized as follows: (1) final diagnosis six months after the initial presentation; (2) how the result of ^18^FDG-PET/CT changed the clinical course; (3) The correlation between the ^18^FDG-PET/CT results and the final diagnosis. In cases that also underwent a pericardial biopsy, the correlation between the pericardial SUV_max_ value and the diagnostic yield of the pericardial biopsy was also determined. Diagnostic results were defined as when a definite diagnosis was able be made from the biopsy results. For example, granulomatous inflammation with caseous necrosis, positive for AFB stain, culture or TB-PCR for tuberculous pericarditis, and malignant cells confirmed regardless of the origin in neoplastic pericarditis. In addition, we collected information on biopsy sites based on an increased ^18^FDG uptake in places other than the pericardium and attempted to determine which site was most frequent and what the yield of a targeted biopsy was.

The PET interpretation of the presence of malignant pericardial lesions was carried out according to the following criteria; (1) probably benign: low or no ^18^FDG uptake in the pericardium without any other organ uptake suspicious for malignancy; (2) equivocal: low or no ^18^FDG uptake in the pericardium with other organ uptake suspicious for malignancy or a previous cancer history; and (3) probably malignant: a high ^18^FDG uptake in the pericardium with other organ uptake suspicious for malignancy.

### Statistical analysis

Categorical variables are presented as the absolute number and the percentage of relative frequencies. Continuous variables are presented as the average and standard deviation (normal distribution) or the median and interquartile range (non-normal distribution) based on normality testing. The Statistical Package for the Social Sciences (SPSS), version 24 (IBM, Armonk, NY, USA) was used for the statistical analysis.

## Abbreviations

AFB: Acid-fasting bacilli
BMI: Body mass index
CMR: Cardiovascular magnetic resonance imaging
CT: Computed tomography
^18^FDG: [18F]2-deoxy-2-fluoro-d glucose
PET: Positron emission tomography
SUV: Standardized uptake value
SUV_max_: Maximum standardized uptake value
TB-PCR: Mycobacterium tuberculosis-polymerase chain reaction
